# Impact of emerging ACA on survival in chronic myeloid leukemia (CML)

**DOI:** 10.1038/s41375-022-01681-3

**Published:** 2022-08-23

**Authors:** Rüdiger Hehlmann, Michael Lauseker, Astghik Voskanyan, Alice Fabarius, Claudia Haferlach, Andreas Hochhaus, Susanne Saußele

**Affiliations:** 1 Medizinische Fakultät Mannheim, Universität Heidelberg, Mannheim, Germany; 2 ELN Foundation, Weinheim, Germany; 3 Institut für Biometrie und Epidemiologie (IBE), Universität München, München, Germany; 4 University of Erivan, Erivan, Armenia; 5 Münchner Leukämielabor, MLL, München, Germany; 6 Klinik für Innere Medizin II, Universitätsklinikum Jena, Jena, Germany

**Keywords:** Translational research, Genetics research

## To the Editor:

We write to expand on our prior publication in Leukemia (Hehlmann et al. High-risk additional chromosomal abnormalities at low blast counts herald death by CML. Leukemia 2020; 34: 2074-86) [1].

Additional chromosomal abnormalities (ACA), in particular high-risk ACA, provide insights into end-phase and progression of chronic myeloid leukemia (CML) and predict survival [2] regardless if they are present at diagnosis [3, 4] or emerge, more frequently, in the course of the disease [1, 5]. ACA which rise from around 10% in chronic phase to up to 90% in blast crisis may be the central mediators of progression.

Not all ACA predict survival equally, but evidence indicates that high-risk ACA do predict a poor prognosis [1, 5, 6]. Currently, high-risk ACA comprise +8, +Ph, i[17q], +19, +17, and +21 (historically called major route [7] because of their frequency in blast crisis of >5%), complex aberrant karyotypes, −7/7q- and 3q26.2 and 11q23 abnormalities. The latter 3 were historically called minor route, because of their frequency in blast crisis of <5% [7]. All other ACA, for the time being, may be called low-risk ACA. There is evidence that also low-risk ACA have a negative impact on survival, though to a much lesser degree than high-risk ACA.

Even amongst high-risk ACA there are differences in impact on prognosis. The prognostic power of ACA depends on the type of ACA, on whether they occur alone or in combination with one or more additional ACA and, possibly, on occurence at diagnosis or later on [1, 5, 6].

Whereas prognostic impact of ACA at diagnosis is readily recognizable by comparison of patients with ACA to patients without ACA, prognostic impact is less readily apparent, if ACA emerge in the course of the disease. If survival of such patients is compared with that of patients without ACA, the lead time from diagnosis to the emergence of ACA should be considered. Otherwise, the prognostic impact of ACA may be biased. Various methodological approaches are possible.

**Fig. 1:**
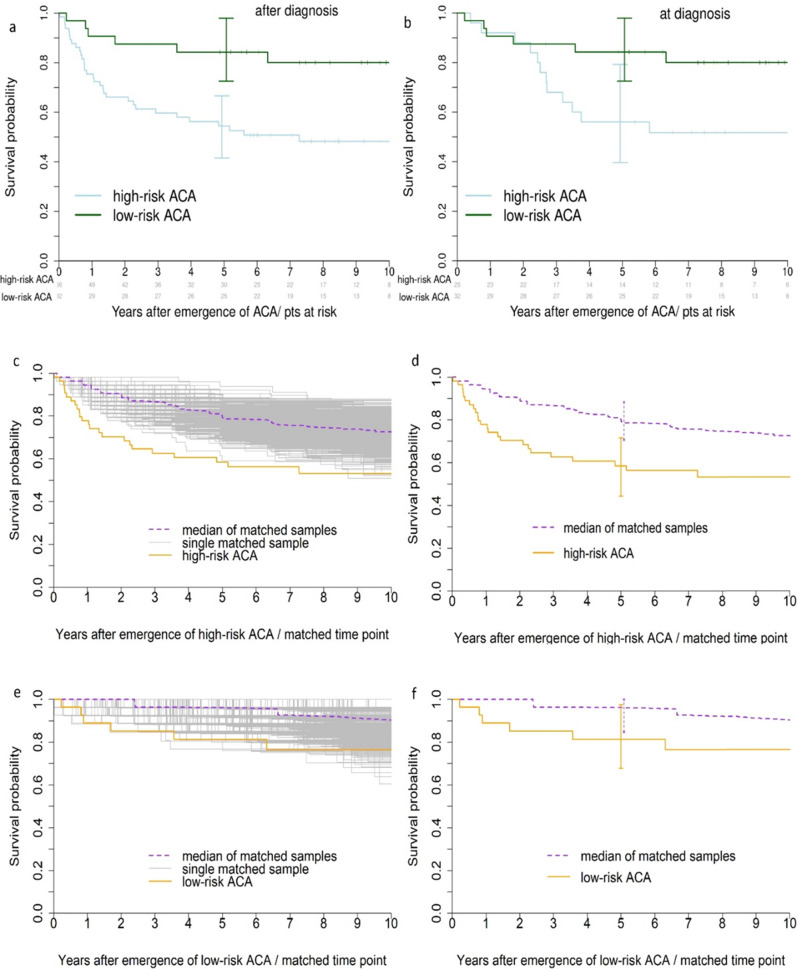
Survival with with high-risk and low-risk ACA occuring at diagnosis or later on. **a** Survival probabilities of CML patients in CP with high-risk ACA emerging after diagnosis (*n* = 66) compared to 32 patients with low-risk ACA occurring either at or after diagnosis synchronized for the time to emergence of ACA. **b** Survival probabilities of patients in CP with high-risk ACA occurring at diagnosis (*n* = 25) compared to the 32 patients with low-risk ACA occurring either at or after diagnosis synchronized for the time to emergence of ACA. Survival with high-risk ACA occuring at versus emerging after diagnosis is not significantly different (*p* = 0.5). **c** Survival probabilities of CML patients in CP with high-risk ACA compared with a matched cohort of patients without high-risk ACA by resampling [9], *p* = 0.024, median hazard ratio 2.15, **d** with confidence intervals. **e** Survival probabilities of patients in CP with low-risk ACA compared with a matched cohort of patients with no ACA by resampling [9], *p* = 0.204, median hazard ratio 2.46, **f** with confidence intervals. CML chronic myeloid leukemia, ACA additional chromosomal abnormalities, pts patients, CP chronic phase.

One way to describe the impact of emerging high-risk ACA on survival correctly is their comparison to patients with low-risk ACA synchronized for the time to emergence of ACA as shown in Fig. 1a, b. This method was used previously to analyse the prognostic impact of emerging ACA in a long-term randomized study [1]. Using this approach, the survival probability of 66 chronic phase CML patients with high-risk ACA emerging after diagnosis (4.4% of 1510 prospectively followed cytogenetically evaluable patients) shown in Fig. 1a is similarly reduced as that of 25 patients with high-risk ACA occurring at diagnosis (1.7%) (Fig. 1b). 32 patients with low-risk ACA occurring at, or emerging after, diagnosis (2.1%) serve as control. Characteristics of patients with high-risk ACA occurring at, or emerging after, diagnosis are comparable (Table 1) except that, in our cohort, patients with ACA emerging after diagnosis are older, in part due to the time elapsed until emergence of ACA, and show, as expected, signs of treatment (cytopenias). Patients are derived from CML study IV [1, 8].

**Table 1:** Patients‘ characteristics at emergence of ACA (*n* = 123).

Time of measurement	with high-risk ACA		with low-risk ACA	
	at diagnosis (*n* = 25)	at emergence after diagnosis (*n* = 66)	at diagnosis (*n* = 19)	at emergence after diagnosis (*n* = 13)
Age (years) - median (max, min)	48 (23–69)	54 (42–89)	48 (18–76)	53 (24–69)
Hb (g/dl) - median (max, min)	11.6 (7.5–5.2)	12.3 (6.4–16.5)	11.1 (8.6–14.4)	11.0 (7.5–13.7)
Anemia - n/valid	7/25	12/46	4/17	2/7
Platelets (/nl) - median (max, min)	328 (82–970)	157 (11–780)	373 (171–1535)	101 (35–350)
Thrombocytopenia (<100.000/ul) - n/valid	0/25	16/46	0/17	2/7
WBC (/nl) - median (max, min)	28.1 (2.0–141)	2.8 (0.1-171)	141.3 (54–359)	7.1 (2.6–84)
Neutropenia (<1000/ul) - n/valid	0/25	11/40	0/17	1/7
Spleen (cm below costal margin) – median (max, min)	0 (0-18)	0 (0–18)	5 (0 – 14)	0 (0–10)
Splenomegaly - n/valid	10/25	10/36	12/17	10/7
Basophils (%) - median (max, min)	3 (0–66)	1 (0–14)	5 (0–15)	0 (0–1)
ELTS Score - n low/int/high	14/5/6	–	9/7/3	–
Time to ACA (months) - median of the observed (max, min) mean of the observed	0 0	15 (1–133) 26	0 0	3 (1–95), 20
n of metaphases assessed - median	20	14	19	8

*WBC* white blood cells, *n* number.

Another way to compare survival of patients with emerging high-risk ACA to patients without high-risk ACA is matching patients with high-risk ACA to patients without high-risk ACA by resampling [9] who (a) lived without high-risk ACA at least until the time point when the high-risk ACA of the partner emerged, (b) had the same age and (c) the same BCR-ABL1 level within + /− 90 days. Matching partners were found for 54 of 66 patients with emerging high-risk ACA. Since for some patients many potential matching partners were found and for others only one, and since individual matching curves looked quite heterogeneous, 1:1 matching was repeated 1000-fold and the median of the resulting curves determined pointwise. The same approach was used for the comparison of patients with low-risk ACA to patients with no ACA. This resampling was done in R 4.0.2.

Figure 1c–f show the survival outcomes by resampling for patients with high-risk (Fig. 1c, d) and with low-risk ACA (Fig. 1e, f), respectively. Figure 1c, e show the curves of all matched samples, the median of the curves, and the survival curve of the patients with high-risk or low-risk ACA, respectively. Figure 1d, f show the respective curves with confidence intervals.

The data demonstrate that high-risk ACA had a negative impact on survival (Fig. 1a–d) and that the great majority of high-risk ACA were not detected at diagnosis, but emerged in the course of disease (72.5%). Under consideration of the lead time to emergence, high-risk ACA emerging after diagnosis seem to confer a poorer prognosis (Fig. 1a) than high-risk ACA detected at diagnosis (Fig. 1b), but this is not significant. Some high-risk ACA may be rarely, or not at all, detectable at diagnosis, emerging mostly in the course of the disease as for instance chromosome 3- or 7-abnormalities [1].

Our data further indicate that also low-risk ACA may have a negative impact on survival if compared to no ACA (Fig. 1e), although the effect did not reach significance (Fig. 1f). Some low-risk ACA, for instance from 6 patients who died during the observation period of a median 9.5 years, may turn out as high-risk ACA in future analyses of larger cohorts. The 6 low-risk ACA are from patients 103, 106, 113, 119, 121, and 123 in appendix A.1 in [1] as follows:

1. 46,XX,t(9;22)(q34;q11)[1]/46,XX,del[5](q13q22),t(9;22)(q34;q11)[24]
2. 46,XX,t(9;22)(q34;q11)[16]/46,XX,t(9;22)(q34;q11),add[20](p11)[9]
3. 46,XY,t(1;9)(q24;q31),t(9;22)(q34;q11)[20]
4. 46,XY,t(6;15),t(9;22)(q34;q11)[6]
5. 46,XX,t(7;7)(p22;q22),t(9;22;9)(q34;q11;p24)[16]
6. 46,XX,t(9;22)(q34;q11)[18]/46,XX,t(9;22)(q34;q11),ins(11;11)(p15;p11.2p13)[4]

There is controversy in the literature about the degree of negative prognostic impact of some high-risk ACA as a single abnormality such as +8 or +Ph [1, 5, 6], but not in combination with other abnormalities. ACA in combination, or complex ACA, as a rule, have higher degrees of negative prognostic impact. Some high-risk ACA are almost exclusively observed in combination, such as +19 [1, 5].

Relating evolving sequencing data [10–12] to cytogenetic data within clinical trials would provide a more comprehensive picture of the relative timing and prognostic impact of genetic abnormalities to better understand the processes underlying progression of CML and to better recognize patients at risk of progression. In the 5th edition of the WHO classification, the accelerated phase of CML has been ommitted in favor of an emphasis on high-risk features such as high-risk ACA. PMID: 35732831.

## References

[1] Hehlmann R, Voskanyan A, Lauseker M, Pfirrmann M, Kalmanti L, Rinaldetti S (2020). High-risk additional chromosomal abnormalities at low blast counts herald death by CML. Leukemia.

[2] Hehlmann R (2020). Chronic myeloid leukemia in 2020. HemaSphere.

[3] Fabarius A, Leitner A, Hochhaus A, Müller MC, Hanfstein B, Haferlach C (2011). Impact of additional cytogenetic aberrations at diagnosis on prognosis of CML: long-term observation of 1151 patients from the randomized CML Study IV. Blood.

[4] Clark RE, Apperley JF, Copland M, Cicconi S (2021). Additional chromosomal abnormalities at chronic myeloid leukemia diagnosis predict an increased risk of progression. Blood Adv.

[5] Wang W, Cortes JE, Tang G, Khoury JD, Wang S, Bueso-Ramos CE (2016). Risk stratification of chromosomal abnormalities in CML in the era of TKI therapy. Blood.

[6] Gong Z, Medeiros LJ, Cortes JE, Chen Z, Zheng L, Li Y (2017). Cytogenetics-based risk prediction of blastic transformation of CML in the era of TKI therapy. Blood Adv.

[7] Johansson B, Fioretos T, Mitelman F (2002). Cytogenetic and molecular genetic evolution of chronic myeloid leukemia. Acta Haematologica.

[8] Hehlmann R, Lauseker M, Saußele S, Pfirrmann M, Krause S, Kolb HJ (2017). Assessment of imatinib as first-line treatment of CML: 10-year survival results of the randomized CML study IV and impact of non-CML determinants. Leukemia.

[9] Simon J. Resampling: the new statistics, 2nd edn. Wadsworth, Boston, 1997

[10] Grossmann V, Kohlmann A, Zenger M, Schindela S, Eder C, Weissmann S (2011). A deep-sequencing study of CML patients in blast crisis (CML-BC) detects mutations in 76.9% of cases. Leukemia.

[11] Branford S, Wang P, Yeung DT, Thomson D, Purins A, Wadham C (2018). Integrative genomic analysis reveals cancer associated mutations at diagnosis of CML in patients with high-risk disease. Blood.

[12] Ochi Y, Yoshida K, Huang Y-J, Kuo M-C, Nannya Y, Sasaki K (2021). Clonal evolution and clinical implications of genetic abnormalities in blastic transformation of chronic myeloid leukaemia. Nat Comm.

